# The role of interpersonal trauma and substance use in mental health: A large population-based study

**DOI:** 10.1016/j.psychres.2023.115712

**Published:** 2024-03

**Authors:** Monica Aas, Lucia Sideli, Christian Franceschini, Luis Alameda, Giulia Trotta, Gianluca Lo Coco, Alessandro Musetti, Adriano Schimmenti

**Affiliations:** a Social, Genetic and Developmental Psychiatry Centre, Institute of Psychiatry, Psychology and Neuroscience, King's College London, London, UK; b Department of Psychosis Studies, Institute of Psychiatry, Psychology & Neuroscience, King's College London, London, England, UK; c Department of Behavioural Sciences, OsloMet – Oslo Metropolitan University, Oslo, Norway; d Department of Human Science, LUMSA University, Rome, Italy; e Department of Medicine and Surgery, University of Parma, Parma, Italy; f Service of General Psychiatry, Treatment and Early Intervention in Psychosis Program, Lausanne, University Hospital (CHUV), Lausanne, Switzerland; g Centro Investigacion Biomedica en Red de Salud Mental (CIBERSAM); Instituto de Biomedicina de Sevilla (IBIS), Hospital Universitario Virgen del Rocio, Departamento de Psiquiatria, Universidad de Sevilla, Sevilla, Spain; h Department of Psychology, Educational Science and Human Movement, University of Palermo, Palermo, Italy; i Department of Humanities, Social Sciences and Cultural Industries, University of Parma, Parma, Italy; j Faculty of Human and Social Sciences, UKE - Kore University of Enna, Enna, Italy

**Keywords:** Substance use, Interpersonal trauma, Mental health, Moderation

## Abstract

Both interpersonal trauma (IPT) and substance use are linked to mental health problems, however their interplay is understudied. This study will investigate the relationship between IPT, substance use and mental health in a large population-based sample.

Participants included 3756 individuals, mainly young university students using a snowball sampling method. History of IPT was collected retrospectively using the Traumatic Experiences Checklist. Substance use was examined using the World Health Organization's Alcohol, Smoking and Substance Involvement Screening Test. Mental health symptoms were assessed by the DSM-5 Level 1 Cross-Cutting Symptom Measure. Moderation analyses were performed investigating the relationship between IPT, substance use, and mental health symptoms.

Participants exposed to IPT had a higher prevalence of substance use (cannabis, alcohol, tobacco) and had more severe mental health problems than people without IPT. Substance use was associated with a blunted increase of depression, anxiety, and anger in trauma victims. A history of abuse was more strongly linked to substance use than neglect. Moderation analyses further revealed that cannabis use increased psychotic symptoms and psychotic symptoms increased cannabis use in participants with high levels of IPT.

Our findings indicate that substance use worsens psychotic symptoms in IPT victims whilst dampening other mental health symptoms.

## Highlights

• Interpersonal trauma is associated with substance use.
• Both interpersonal trauma and substance use were associated with mental health symptoms.
• A positive bidirectional relationship was observed between cannabis use and psychosis symptoms in trauma victims.

## 1 Introduction

Interpersonal trauma (IPT) and substance use are associated with increased risk of psychopathology, cutting across affective and non-affective diagnosis (Radua et al., 2018; Rodriguez et al., 2021; van Nierop et al., 2015). The literature supports that IPT and substance use often co-occur in people with mental problems (Aas et al., 2014; Etain et al., 2013; Khoury et al., 2010). Understanding if the use of common substances (such as alcohol, cannabis or tobacco) worsen mental symptoms in people with a trauma history is yet to be clarified. Existent studies tend to rely on small clinical research with limited statistical power.

IPT is associated with risk of addictive behaviours (Konkolÿ Thege et al., 2017), including alcohol (Berenz et al., 2016; Shin et al., 2009; Thompson et al., 2008), tobacco (Kuzminskaite et al., 2021; Shin, 2021), cannabis abuse (Aas et al., 2014; Chu, 2012), and substance poly-abuse (Hedtke et al., 2008; Schimmenti et al., 2022). These findings are observed in both general population (Hicks et al., 2022) and clinical samples (Trotta et al., 2023; Aas et al., 2014). Yet, the interplay between IPT, substance use, and mental health symptoms is still understudied. Possible moderation effects may be present between trauma exposure, substance use and mental health symptoms depending on type of symptom. For example, cannabis use may reduce depressive symptoms but increase psychotic symptoms in vulnerable individuals (Di Forti et al., 2019; Feingold and Weinstein, 2021; Quattrone et al., 2019). Recent studies showed that the use of cannabis mediates the link between some traumatic events and the risk of psychosis (Frydecka et al., 2020; Trotta et al., 2023) which suggest that for some individuals the impact of IPT on severe mental health symptoms is via cannabis use (Frydecka et al., 2020; Trotta et al., 2023). Furthermore, the recent legalization of cannabis across western countries (Di Forti, 2020) highlights the pivotal importance to understand the impact of substance use on mental health among vulnerable individuals.

The effect of traumatic events on mental health may be more profound when trauma is intentionally caused by other people known to the victim. This is defined IPT and include emotional, physical, and sexual abuse, physical and emotional neglect, and other types of interpersonal violence (Gilbert et al., 2009; Konkolÿ Thege et al., 2017; López-Martínez et al., 2018). In addition, the type of IPT may be of importance. For instance, Salokangas et al. (2018) found that, compared to emotional and physical neglect and to emotional abuse, sexual and physical abuse has a direct effect on alcohol misuse and a mediating effect, through depressive symptoms. However, the study by Salokangas and colleagues (*N* = 690) only investigated depressive symptoms and alcohol use without taking in consideration other types of substances or mental health symptoms. Furthermore, Yoon et al. (2020) found that physical abuse is associated with a more rapid increase in the risk for tobacco smoking, whereas neglect with a more gradual increase of smoking following childhood maltreatment. However, the study by Yoon and colleagues (comprised of 903 individuals) only investigated cigarette smoking and no other forms of tobacco or substances. To date, large population-based studies are lacking investigating whether the use of socially accepted substances, such as tobacco (e-cigarettes and cigarettes), alcohol, and cannabis, may moderate the effect of IPT on mental health symptoms in the general population across mental health symptoms and trauma subtypes.

The aim of this study was to investigate the relationship between various types of IPT, mental health symptoms and use of tobacco (e-cigarettes and cigarettes), alcohol, and cannabis in a large (*N* = 3756) population-based sample. We hypothesize that (1) a history of IPT will be associated with higher use of common substances, including cannabis, alcohol, and tobacco (e-cigarettes and cigarettes); (2) the relationship between substance use and mental health symptoms will vary in direction depending on the type of symptoms. Consistently with previous studies (Feingold and Weinstein, 2021; Quattrone et al., 2019), we specifically hypothesize for psychotic symptoms that (3) a bidirectional relationship will be observed, i.e., that cannabis use will increase psychotic symptoms, and that high symptom load will predict higher cannabis use. By contrast, we hypothesize (4) a more blunted bidirectional association between cannabis use and depressive and anxiety symptoms.

## 2 Methods

### 2.1 Participants and procedure

Data were collected from general population following a snowball sampling method. 3756 participants were included in the study. Participants were requested to complete a 20-min online survey that was advertised via university communication systems as well as social media. Furthermore, respondents were asked to spread the survey among acquaintances, see Sideli et al. (2023) for more details). Participants signed the electronic informed consent after obtaining general information about the aim of the study. Exclusion criteria were age under 18 and not having Italian as first language. Participation was confidential and voluntary, and all participants could withdraw from the study at any time. Anonymity was guaranteed, as no data on the participants’ identification, or their Internet Protocol address, were collected. The Parma University Ethics committee approved this study (prot. N. 184,304). The study was conducted in accordance with the Declaration of Helsinki (World Medical, 2001) and the ethical guidelines for psychological research laid down by the Italian Psychological Association (AIP) (Associazione Italiana di Psicologia, 2022, https://aipass.org/wp-content/uploads/2023/02/Codice-Etico_luglio-2022.pdf).

### 2.2 Measures

The World Health Organization's Alcohol, Smoking and Substance Involvement Screening Test (ASSIST) (Humeniuk et al., 2006) evaluates substance addictive behaviours related to tobacco, alcohol, and cannabis use. In this study, we employed a modified version of the ASSIST also including electronic smoking devices (i.e., e-cigarettes). The questionnaire assesses the frequency and consequences of substance addictive behaviours during the previous three months (e.g., “During the past three months, how often has your use of [the substance] led to health, social, legal or financial problems?”) and rated on a scale from 0 (“Never”) to 7 (“Almost daily/ daily”). The ASSIST has demonstrated a good discriminant validity in several countries (Humeniuk et al., 2008; Newcombe et al., 2005). In this study, the internal validity (estimated by Cronbach's alpha) was 0.81 for tobacco, 0.75 for e-cigarettes, 0.70 for alcohol, and 0.76 for cannabis.

The Traumatic Experiences Checklist (TEC) (Nijenhuis et al., 1999; Italian version by Schimmenti, 2018) is a 29-item retrospective self-report questionnaire examining 29 types of lifetime potential traumatic events (e.g., “When you were a child or a teenager have you ever felt emotionally neglected (e.g., being left alone, insufficient affection) by your parents, brothers or sisters?”). For the purposes of this study, a total TEC score was calculated by summing the number of relevant events that were experienced (absent = 0, present = 1). The TEC showed a good test–retest reliability (Nijenhuis et al., 2002), as well as adequate internal reliability, construct validity and convergent validity with reports of traumatic experiences (Craparo et al., 2013; Schimmenti, 2018). In the current study, the KR-20 coefficient for the TEC was 0.72. In order to analyze the relationship between substance use, psychopathological symptoms, and specific types of IPT, the following subscales were used with three items per subscale: emotional neglect, emotional abuse, physical abuse, physical bodily threat, sexual harassment, and sexual abuse.

The DSM-5 Level 1 Cross-Cutting Symptom Measure (DSM-XC) (American Psychiatric Association, 2013; Italian version by Fossati et al., 2015) is a 23-item self-report questionnaire assessing psychopathological symptoms in the previous two weeks. The DSM-XC assesses the following clinical domains: depression, anger, mania, anxiety, somatic symptoms, suicidal ideation, psychosis, sleep problems, memory, repetitive thoughts and behaviours (i.e., obsessive – compulsive), dissociation, substance use, and impaired personality functioning (i.e., impairment in self and interpersonal functioning). A sample item is “During the past two weeks, how much (or how often) have you been bothered by feeling down, depressed, or hopeless?”. Participants were requested to respond on a 5-point Likert scale, from 0 (“Not at all”) to 4 (“Nearly every day”). We computed mean scores for each clinical domain and for the total scale. The DSM-XC has a good convergent validity with psychopathology measures (Bravo et al., 2018). In this study, the internal consistency of the total scale (Cronbach's alpha value) was 0.86.

### 2.3 Statistical analyses

Spearman's correlations were used to investigate the association between IPT total score and subtypes and substance use (cannabis, alcohol, and tobacco). The SPSS Process macro by Hayes (2013) with 5000 bootstrapping was used to test the moderated models in which (1) symptom severity moderated the association between reports of IPT and substance use (cannabis, alcohol, tobacco, and e-cigarettes); and (2) substance use moderated the association between reports of IPT and symptoms severity, see Supplementary Material Figs. 1, and 2. To test the moderating effect of symptom severity on the relationship between IPT and substance use, and the moderating effect of substance use on the relationship between IPT and symptom severity, moderating variables were categorized as “low” symptoms/ “low” substance use “(i.e., below −1 standard deviation (SD) the mean of the sample distribution); “mean” symptoms/ “mean” substance use “(i.e., within −1 and + 1 SD of the mean); and “high” symptoms/ “high” substance use “(i.e., above +1 SD of the sample distribution).

To mitigate concerns associated with multiple testing, our initial analyses involved employing the total trauma score. Additionally, we examined distinct categories of trauma (i.e., emotional neglect, emotional abuse, physical abuse, bodily threat, sexual harassment, and sexual abuse) separately, but only when the total trauma score yielded statistically significant results. Secondly, in order to conduct sub-analyses focused on specific trauma subtypes, the p-value threshold was adjusted for number of trauma types (that is, 0.05/6 = 0.008). Analyses were run using the Statistical Package for the Social Sciences (SPSS) program version 27.0.

## 3 Results

### 3.1 Descriptive overview of the sample

The mean age of the sample was 23.8 (SD = 3.53). The majority of the sample was female (78 %). Most of the sample were students (74.5 %; see Table 1 for details).

**Table 1:** Descriptive overview of the sample (*N* = 3756).

Age, mean ± SD	23.84 ± 3.53
Sex, N (%)	
Females	2931 (78.0)
Males	825 (22.0)
Occupation, N (%)	
Student	2797 (74.5)
Employee worker	612 (16.3)
Unemployed	178 (4.7)
Self-employed	169 (4.5)
TEC trauma, mean ± SD, (min-max)	
Total score	2.39 ± 2.42 (0–16)
Emotional neglect	.87 ± .97 (0–3)
Emotional abuse	.69 ± .82 (0–3)
Physical abuse	.17 ± .46 (0–3)
Physical bodily threat	.40 ± .61 (0–3)
Sexual harassment	.17 ± .40 (0–3)
Sexual abuse	.09 ± .32 (0–3)
Cannabis, mean ± SD (min-max)	2.50 ± .10.65 (0–38)
Tobacco (electric), mean ± SD	1.83 ± 4.70 (0–35)
Tobacco (cigarettes), mean ± SD	9.08 ± 10.65 (0–57)
Alcohol use, mean ± SD	6.88 ± 6.32
Depression, mean ± SD	2.05 ± 1.06
Anger, mean ± SD	2.14 ± 1.18
Mania, mean ± SD	1.52 ± .97
Anxiety, mean ± SD	1.93 ± .97
Somatic, mean ± SD	1.46 ± 1.11
Suicide, mean ± SD	.68 ± .85
Psychosis, mean ± SD	.58 ± .70
Sleep, mean ± SD	1.64 ± 1.27
Memory, mean ± SD	1.01 ± 1.07
Repetitive, mean ± SD	1.07 ± .99
Dissociation, mean ± SD	1.05 ± 1.10
Dysfunctional personality traits, mean ± SD	1.69 ± 1.16
Substance use, mean ± SD	1.03 ± .93
General, mean ± SD	1.37 ± .67

TEC= The Traumatic Experiences Checklist.

### 3.2 Interpersonal trauma events and substance use

Individuals reporting having IPT were more likely to use cannabis (rho = 0.14, *p* < .001), consume more alcohol units (rho=0.12, *p* < .001), tobacco cigarettes (rho = 0.13, *p* < .001), and e-cigarettes (rho = 0.08, *p* < .001, see Table 2). Dividing into subtypes of IPT, physical abuse was the subtype most associated with cannabis (rho = 0.13, *p* < .001) and alcohol use (rho = 0.10, *p* < .001), whilst emotional neglect and physical abuse were most commonly reported in tobacco smokers (cigarettes) (rho = 0.12, *p* < .001), and emotional neglect with e-cigarettes users (rho = 0.08, *p* < .001).

**Table 2:** Childhood trauma and substance use.

Spearman's Correlation	Childhood trauma total score
Cannabis	*r* = 0.14, *p* < .001
Alcohol	*r* = 0.12, *p* < .001
Tobacco, cigarettes	*r* = 0.13, *p* < .001
Tobacco, electric	*r* = 0.08, *p* < .001

### 3.3 Mental health symptoms as a moderator between traumatic experiences and substance use

#### 3.3.1 Cannabis

**Fig. 1:**
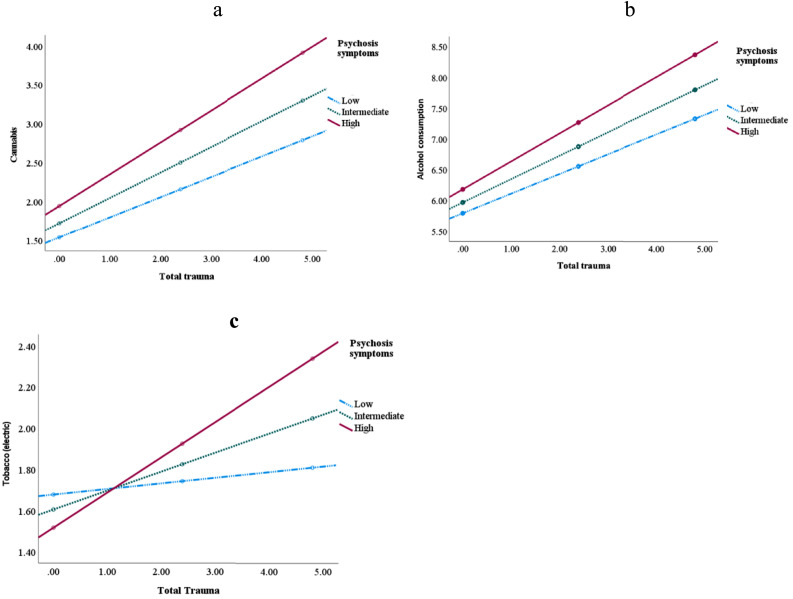
a-c. Psychosis symptoms moderated the association between interpersonal trauma and substance use. *Process,* Psychosis symptoms moderated the associations between interpersonal trauma and substance use. “Low” = −1 SD mean symptoms; “Intermediate” = symptoms at mean level; and “high” = symptoms 1 SD above mean.

Psychosis symptoms moderated the associations between IPT and cannabis use (β = 0.12, *p* = .005, Fig. 1a). A trend was observed for sleep problems and general symptoms (β = 0.05, *p* = .07 and β = 0.08, *p* = .09, respectively, see Table 3). Mental health symptoms (psychosis symptoms, poor sleep, and general symptoms) moderated the association between abuse and cannabis use but not neglect (see Supplementary Material, Table S1). No moderation effects were observed for depressive symptoms, anger, mania, anxiety, somatic symptoms, suicidal symptoms, memory, repetitive symptoms, dissociation, or dysfunctional personality traits (all ps>05).

**Table 3:** Mental health symptoms as a moderator between cannabis use and total trauma.

	Psychosis symptoms	Sleep symptoms	General symptoms
	ß, t, se, p	ß, t, se, p	ß, t, se, p
**Total trauma** Interaction:	.12, 2.78, 0.04, 0.005	.05, 1.80, 0.03, 0.07	.08, 1.70, 0.05, 0.09
Conditional Effect: High symptoms	.41, 9.47, 0.04, <.001	.36, 8.09, 0.05, <.001	.27, 6.40, 0.04, <.001
Intermediate symptoms	.33, 8.95, 0.04, <.001	.30, 7.77, 0.04, <.001	.21, 5.31, 0.04, <.001
Low symptoms	.26, 5.54, 0.05, <.001	.24, 4.02, 0.05, 0.0001	.16, 2.60, 0.06, 0.008

Process, moderation analysis.

#### 3.3.2 Alcohol

For psychosis symptoms, an interaction was observed between IPT and alcohol consumption (β = 0.11, *p* = .03; see Table 4, Fig. 1b). Lastly, psychotic symptoms moderated the association between abuse (physical, emotional, and sexual abuse) and alcohol consumption but not neglect (see Supplementary Material, Table S2).

**Table 4:** Mental health symptoms as a moderator between alcohol and total trauma.

	Psychosis symptoms	Anxiety symptoms
	ß, t, se, p	ß, t, se, p
**Total trauma** Interaction:	.11, 2.17, 0.05, 0.03	−0.08, −1.88, 0.04, 0.06
Conditional Effect: High symptoms	.46, 9.03, 0.05, <.001	.32, 5.98 0.04, <.001
Intermediate symptoms	.38, 8.94, 0.05, <.001	.40, 8.89, 0.04, <.001
Low symptoms	.32, 5.85, 0.05, <.001	.47, 6.91, 0.04, <.001

#### 3.3.3 Tobacco

Psychotic symptoms and dysfunctional personality traits moderated the relationship between traumatic experiences and e-cigarettes use (β = 0.11, *p* = .002; β = 0.06, *p* = .03, respectively, please see Table 5, Fig. 1c). Specifically, psychotic symptoms moderated the association between abuse (physical, emotional, and sexual abuse) and e-cigarettes use but not neglect (see Supplementary Material, Table S3). Similarly, dysfunctional personality traits moderated the association between abuse (emotional and physical abuse) and e-cigarettes use, but not neglect.

**Table 5:** Mental health symptoms as a moderator between tobacco use (electric) and total trauma.

	Psychosis symptoms	Dysfunctional personality traits
	ß, t, se, p	ß, t, se, p
**Total trauma** Interaction:	.11, 3.06, 0.04, 0.002	.06, 2.21, 0.03, 0.03
Conditional Effect: High symptoms	.17, 4.51, 0.04, <.001	.15, 3.67, 0.04, 0.0002
Intermediate symptoms	.09, 0.29, 0.03, 0.004	.08, 0.23, 0.03, 0.02
Low symptoms	.03, 0.66, 0.04, 0.51	.01, 0.15, 0.05, 0.88

Process, moderation analysis.

### 3.4 Substance use as moderators between traumatic experiences and mental health symptoms

#### 3.4.1 Cannabis

**Fig. 2:**
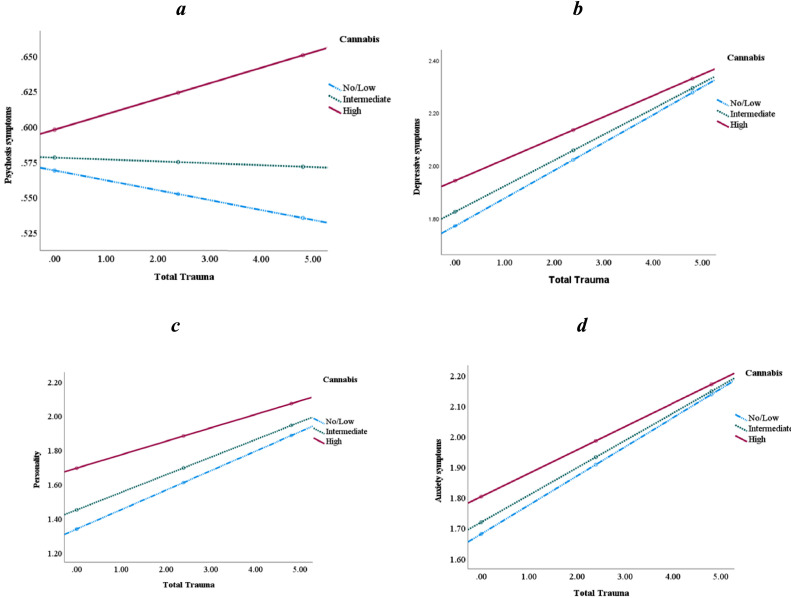
a-d Cannabis use moderated the association between interpersonal trauma and depressive, anxiety symptoms and dysfunctional personality traits. *Process,* Cannabis use moderated the associations between interpersonal trauma and mental health symptoms. “Low” = −1 SD mean symptoms; “intermediate” = symptoms at mean level; and “high” = symptoms 1 SD above mean.

Cannabis moderated the association between IPT and psychotic symptoms, in the direction of participants with high cannabis use had the most psychotic symptoms if exposed to high levels of trauma (β = 0.002, *p* = .001, Fig. 2a). On the other hand, cannabis use negatively moderated the association between IPT and depressive symptoms, having dysfunctional personality traits and anxiety symptoms, so that the effect of IPT on symptom severity was greater among those with low cannabis use, as compared to those with mean and high cannabis use (please see Table 6, Fig. 2b-d). Cannabis moderated the relationship between symptoms across types of abuse and neglect (see Supplementary Material, Table S4).

**Table 6:** Cannabis use as a moderator between mental health symptoms and total trauma.

	Depressive symptoms	Anxiety symptoms	Suicide symptoms
	ß, t, se, p	ß, t, se, p	ß, t, se, p
**Total trauma** Interaction:	−0.003, −3.03, 0.001, 0.003	−0.002, −2.48, 0.001, 0.01	.001, 1.73, 0.001, 0.08
Conditional Effect: High symptoms	.08, 9.63, 0.01, <.001	.08, 9.98, 0.01, <.001	.06, 8.81, 0.01, <.001
Intermediate symptoms	.10, 13.68, 0.01, <.001	.09, 13.67, 0.01, <.001	.05, 8.97, 0.01, <.001
Low symptoms	.11, 13.26, 0.01, <.001	.10, 13.07, 0.01, <.001	.05, 7.48, 0.01, <.001
	**Psychosis symptoms**	**Dysfunctional personality traits**	
	ß, t, se, p	ß, t, se, p	
**Total trauma** Interaction:	.002, 3.23, 0.001, 0.001	−0.005, −3.99, 0.001, 0.0001	
Conditional Effect: High symptoms	.01, 1.93, 0.01, 0.05	.08, 8.70, 0.01, <.001	
Intermediate symptoms	−0.001, −0.27, 0.01, 0.78	.10, 13.34, 0.01, <.001	
Low symptoms	−0.01, −1.29, 0.01, 0.20	.11, 13.27, 0.01, <.001	

Process, moderation analysis.

#### 3.4.2 Alcohol

**Fig. 3:**
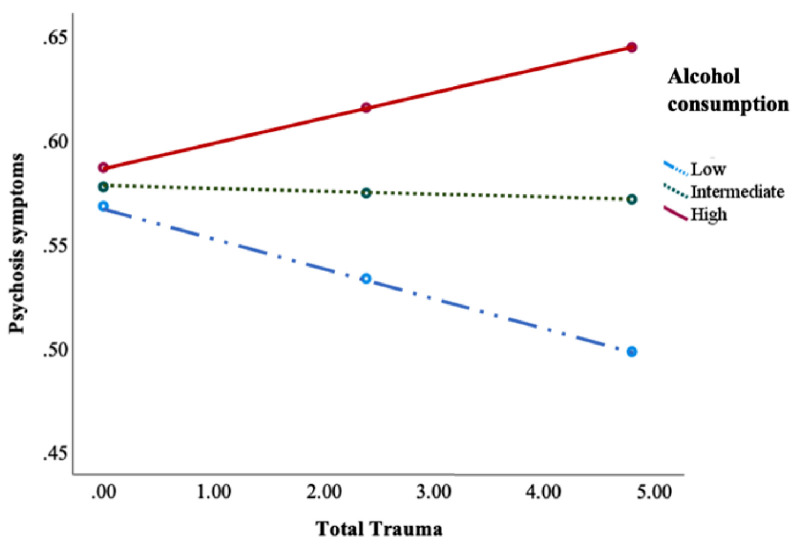
Alcohol use moderated the association between interpersonal trauma and psychotic symptoms. *Process,* Alcohol use moderated the associations between interpersonal trauma and dysfunctional personality traits. “Low” = −1 SD mean symptoms; “intermediate” = symptoms at mean level; and “high” = symptoms 1 SD above mean.

Alcohol use moderated the association between IPT and psychotic symptoms, suicide ideation and memory problems (see Fig. 3, Table 7). Alcohol consumption also negatively moderated the association between IPT and depressive, anger, anxiety and somatic symptoms as well as having a more dysfunctional personality trait (see Table 7). Dividing into subtypes of IPT showed associations across subtypes of trauma but displayed the most consistent findings for physical and emotional abuse (see Table S5).

**Table 7:** Alcohol use as a moderator between mental health symptoms and total trauma.

	Depressive symptoms	Anger symptoms	Anxiety symptoms
	ß, t, se, p	ß, t, se, p	ß, t, se, p
**Total trauma** Interaction:	−0.003, −3.05, 0.001, 0.002	−0.002, −2.06, 0.001, 0.04	−0.003, 2.96, 0.01, 0.003
Conditional Effect: High symptoms	.08, 9.47, 0.001, <.001	.10, 9.15, 0.01, <.001	.08, 9.85, 0.01, <.001
Intermediate symptoms	.10, 13.48, 0.01, <.001	.09, 11.17, 0.01, <.001	.09, 13.84, 0.007, <.001
Low symptoms	.11, 11.35, 0.01, <.001	.08, 8.18, 0.01, <.001	.11, 5.58, 0.01, <.001
	**Somatic symptoms**	**Suicidal ideations**	**Psychosis symptoms**
	ß, t, se, p	ß, t, se, p	ß, t, se, p
**Total trauma** Interaction:	−0.002, −2.55, 0.001, 0.01	.002, 2.82, 0.001, 0.005	.002, 3.35, 0.01, 0.0008
Conditional Effect: High symptoms	.10, 12.00, 0.01, <.001	.07, 9.65, 0.01, <.001	.01, 2.12, 0.01, 0.03
Intermediate symptoms	.12, 16.01, 0.01, <.001	.05, 9.18, 0.01, <.001	−0.001, −0.26, 0.005, 0.79
Low symptoms	.14, 12.87, 0.01, <.001	.04, 4.77, 0.01, <.001	−0.01, −2.13, 0.007, 0.03
	**Memory symptoms**	**Dysfunctional personality traits**	
	ß, t, se, p	ß, t, se, p	
**Total trauma** Interaction:	.002, 1.76, 0.01, 0.08	−0.003, −2.55, 0.001, 0.01	
Conditional Effect: High symptoms	.09, 10.64, 0.01, <.001	.09, 9.64, 0.01, <.001	
Intermediate symptoms	.08 10.27, 0.01, <.001	.10, 13.27, 0.01, <.001	
Low symptoms	.07, 6.76, 0.01, <.001	.12, 10.92, 0.01, <.001	

Process, moderation analysis.

#### 3.4.3 Tobacco

**Fig. 4:**
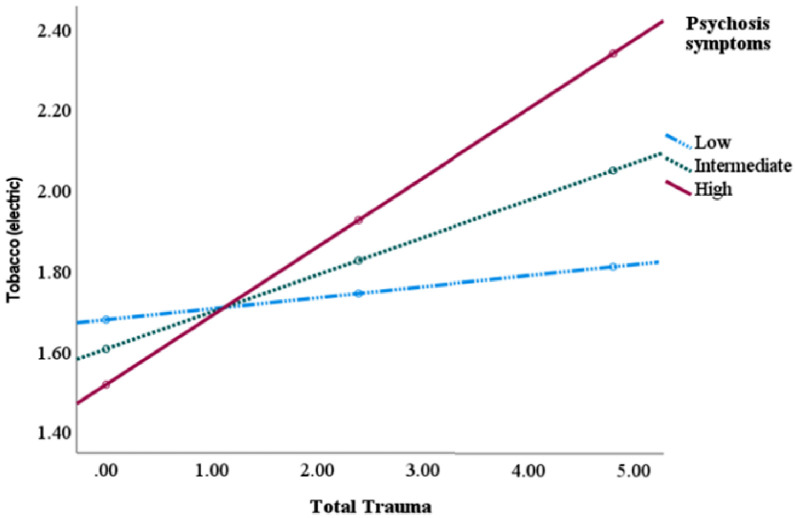
Tobacco use moderated the association between interpersonal trauma and psychotic symptoms. *Process,* Tobacco use moderated the associations between interpersonal trauma and psychotic symptoms. “Low” = −1 SD mean symptoms; “intermediate” = symptoms at mean level; and “high” = symptoms 1 SD above mean.

Tobacco (e-cigarettes) moderated the association between IPT and suicide attempts (β = 0.003, *p* = .02), psychosis symptoms (β = 0.004, *p* = .0001), and poorer memory (β = 0.003, *p* = .02, please see Table 8, Fig. 4). Tobacco (cigarettes) moderated the association between IPT and psychosis symptoms (β = 0.001, *p* = .005), and repetitive behavior (β = 0.001, *p* = .03, please see Table 8). Dividing into subtypes of IPT showed that the slope of poor mental health was the most severe for those using e-cigarettes and exposed to sexual abuse whilst no associations were observed for neglect (See Supplementary material, Table S6). For tobacco cigarettes, similar findings as for e-cigarettes was observed for psychotic symptoms whilst for repetitive behaviors this was observed for neglect and physical abuse but not sexual abuse.

**Table 8:** Tobacco as a moderator between mental health symptoms and total trauma.

Electric	Suicide symptoms	Psychosis symptoms	Memory
	ß, t, se, p	ß, t, se, p	ß, t, se, p
**Total trauma** Interaction:	.003, 2.25, 0.001, 0.02	.004, 4.05, 0.001, 0.0001	.003, 2.36, 0.001, 0.02
Conditional Effect: High symptoms	.07, 9.27, 0.01, <.001	.02, 3.33, 0.01, 0.0009	.10, 10.79, 0.01, <.001
Intermediate symptoms	.06, 10.09, 0.01, <.001	.003, 0.68, 0.004, 0.50	.09, 11.99, 0.01, <.001
Low symptoms	.05, 8.59, 0.01, <.001	−0.004, −0.70, 0.01, 0.48	.08, 10.32, 0.01, <.001
	**Repetition**	**Dysfunctional personality traits**	
	ß, t, se, p	ß, t, se, p	
**Total trauma** Interaction:	.002, 1.83, 0.001, 0.07	.003, 1.89, 0.002, 0.06	
Conditional Effect: High symptoms	.10, 11.42, 0.01, <.001	.12, 12.01, 0.01, <.001	
Intermediate symptoms	.09, 13.30, 0.01, <.001	.11, 14.01, 0.01, <.001	
Low symptoms	.08, 11.70, 0.01, <.001	.10, 12.33, 0.01, <.001	
**Cigarettes**	**Anger symptoms**	**Psychosis symptoms**	**Repetition**
	ß, t, se, p	ß, t, se, p	ß, t, se, p
**Total trauma** Interaction:	−0.01, −1.68, 0.001, 0.09	.001, 22.81, 0.0004, 0.005	.001, 2.14, 0.001, 0.03
Conditional Effect: High symptoms	.07, 7.16, 0.01, <.001	.01, 2.04, 0.01, 0.04	.10, 11.09, 0.01, <.001
Intermediate symptoms	.09, 10.17, 0.01, <.001	−0.01, −1.16, 0.01, 0.24	.08, 9.90, 0.01, <.001
Low symptoms	.10, 9.09, 0.01, <.001	−0.01, −1.69, 0.01, 0.09	.07, 7.90, 0.01, <.001

Process, moderation analysis.

## 4 Discussion

The results of the current study support that individuals exposed to IPT have more severe mental problems and a higher prevalence of substance use (cannabis, alcohol, tobacco) than people without IPT history. Importantly, we found that using cannabis increased psychotic symptoms in traumatized individuals, whilst cannabis users had a slower increase slope of depressive symptoms, anxiety symptoms and dysfunctional personality traits than individuals with no use. Psychotic symptoms were the only mental health symptoms showing a positive bidirectional relationship with substance use including cannabis, alcohol and tobacco use. Thus, substance use steadily increased psychotic symptoms in vulnerable individuals whereas we did not find the same for other types of mental health symptoms.

People with mental health issues have shorter life expectancy than people with a more robust mental health. For example, the lifespan of people with bipolar disorder (BD) is ten to fifteen years shorter than the general population (Hayes and Rockwood, 2019, Laursen, 2011), with similar or even more severe findings in schizophrenia, depression, and substance use disorders (Wildgust et al., 2010). People with mental health issues are also more prone to somatic diseases such as cardiovascular diseases and dementia (Hayes and Rockwood, 2019; Launders et al., 2022). Important contributors are modifiable factors, including lifestyle factors such as substance use (Firth et al., 2020). Our study contributes with new knowledge highlighting the importance of targeting both substance use and trauma-related sequelae as an opportunity to help individuals’ health issues, in addition to the traditional treatment of the symptoms themselves.

Our findings are supported by previous studies showing that both IPT and cannabis use are linked to increased risk of psychopathology, by increased frequency and severity of clinical characteristics (Aas and Etain, 2020, Alameda et al., 2021, Etain and Aas, 2021, Quattrone et al., 2019). An underlying biological mechanism explaining the relationship between IPT and substance use may be their opposite effects on the hypothalamic pituitary adrenal (HPA) axis (Aas et al., 2014). Cannabis and substance use have been linked to a reduction in HPA activity (van Leeuwen et al., 2011), whilst the HPA axis is increased in people with a trauma history, both in depression (Heim et al., 2008) and in psychosis (Aas et al., 2019). Thus, it could be hypothesized that substance use in individuals with a history of IPT may be viewed as a way of “regulating” the HPA axis, which is consistent with the seminal self-medication theory (Khantzian, 1997; Schimmenti et al., 2022) suggesting that individuals may resort to specific substances to artificially regulate their negative emotions. Yet, this in some cases can generate a vicious cycle wherein an increased substance use increases clinical symptoms.

Accordingly, apart from the stimulating Tetrahydrocannabinol (THC), cannabis and cannabis resin contain many other cannabinoids. One of these (cannabidiol, CBD) has attracted recent interest because it is believed to show mood stabilizing properties (Home Office Cannabis Potency Study, 2008, https://lx.iriss.org.uk/content/home-office-cannabis-potency-study-2008.html). It has been suggested that the CBD part of cannabis has a depressive and anxiety dampening effects (García-Gutiérrez et al., 2020) which could explain the blunted slope in symptoms following cannabis use. Whether CBD may be a potential treatment target for people with early trauma needs further investigation.

The findings of the present study should be interpreted considering several limitations. First, a history of IPT was assessed retrospectively using the TEC. Although TEC is a validated measure of trauma, retrospective measures of trauma may be prone to recall bias and do not entirely overlap with prospective measures (Baldwin et al., 2019). Second, several factors should be considered when investigating cannabis use, specifically the potency, frequency, and duration. For example, THC/CBD levels vary in the different types of cannabis, with synthetic skunk showing the highest THC levels, compared to the herbal version. Future studies on the relationship between IPT, substance use, and symptom severity should account for substance potency. Furthermore, our results rely on cross-sectional data, therefore causal conclusion cannot be drawn. Lastly, participants were mostly Italian speaking college students recruited through online survey advertised via university communication systems and social media following a snowball sampling method. This may have affected the general representativeness of the population. Multiple large-scale surveys highlight ethnic and socio demographic disparities in rates of drug use and related negative consequences (Grant et al., 2016). Thus, implementing research studies which takes into account ethnic or racial disparities and socio-economic factors would be of importance.

## 5 Conclusion

People with IPT are more prone to substance use compared to individuals without trauma. Clinical implications of the positive bidirectional relationship of cannabis and alcohol use in people with IPT indicate that these substances exaggerate and worsen specific mental health issues in the general population. As it is a trend for legalizing substances across countries and thus potentially easier accessed, our results indicate the importance of increasing public awareness of psychotic risk linked to their use in vulnerable individuals.

## Financial disclosure

Dr Aas is funded by the MRC (#MR/W027720/1).

## Author statement

All authors contributed to this Manuscript.

## CRediT authorship contribution statement

**Monica Aas:** Formal analysis, Writing – original draft, Writing – review & editing, Visualization. **Lucia Sideli:** Project administration, Writing – original draft, Writing – review & editing, Conceptualization, Formal analysis, Investigation, Validation, Visualization. **Christian Franceschini:** Writing – original draft, Writing – review & editing. **Luis Alameda:** . **Giulia Trotta:** Writing – original draft, Writing – review & editing. **Gianluca Lo Coco:** Writing – original draft, Writing – review & editing. **Alessandro Musetti:** Conceptualization, Data curation, Formal analysis, Funding acquisition, Investigation, Methodology, Project administration, Resources, Software, Supervision, Validation, Visualization, Writing – original draft, Writing – review & editing. **Adriano Schimmenti:** Conceptualization, Data curation, Formal analysis, Funding acquisition, Investigation, Methodology, Project administration, Resources, Software, Supervision, Validation, Visualization, Writing – original draft, Writing – review & editing.

## Declaration of competing interest

Nothing to disclose.

## Appendix Supplementary materials

Image, application 1
