# Supplementary material for: The role of interpersonal trauma and substance use in mental health: A large population-based study
Source: Psychiatry Res. 2024 Mar;333:115712. doi: 10.1016/j.psychres.2023.115712 (PMC11137873; doi:10.1016/j.psychres.2023.115712)
Supplement: Supplementary file 1 [file mmc1.docx]

**Supplementary Material**

**Figure S1, S2.** Diagram Moderation analysis in *Process*

**Fig. S1.**


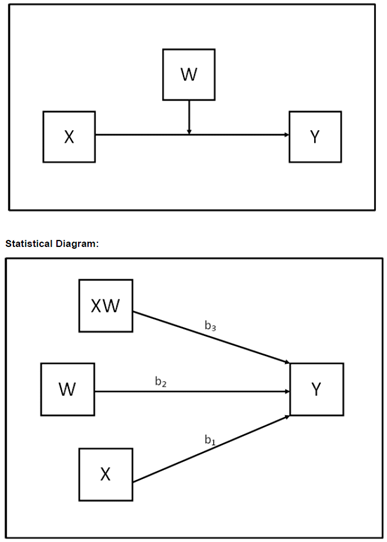


The Predictor (X) is interpersonal trauma (IPS); symptom severity (W) and the outcome (Y) is substance use

**Fig. S2.**


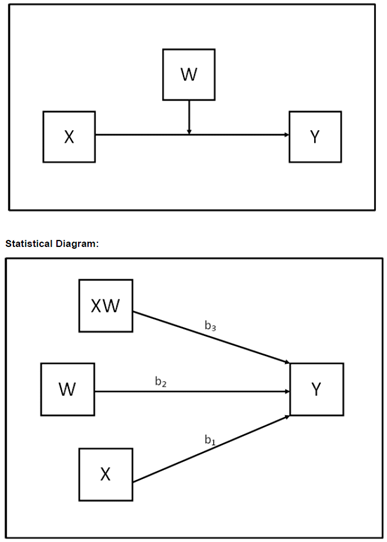


The Predictor (X) is interpersonal trauma (IPS); substance use (W) and the outcome (Y) is symptom severity

**Table S1.** Mental health symptoms as a moderator between cannabis use and subtypes of trauma

|  | **Psychosis symptoms** | **Sleep symptoms** | **General symptoms** |
| --- | --- | --- | --- |
|  | ß, t, se, p | ß, t, se, p | ß, t, se, p |
| **Emotional Neglect**  Interaction: | n.s. | n.s. | n.s. |
| **Emotional abuse**  Interaction: | .30,3.47, .13, .005 | .19, 2.23, .08, .03 | .45, 3.01, .15, .003 |
| Conditional Effect:  High symptoms | .97, 7.05, .13, <0.001 | .77, 5.55, .14, <0.001 | .60, 4.60, .13, <0.001 |
| Intermediate symptoms | .63, 5.93, .63, <0.001 | .53, 4.79, .11, <0.001 | .30, 2.65, .11, .008 |
| Low symptoms | .36, 2.62, .13, .009 | .30, 1.77, .17, .08 | -.004, -.02, .17, .98 |
| **Physical abuse**  Interaction: | .71, 3.40, .21, .0007 | .30, 2.05, .15, .04 | .48, 1.86, .26, .06 |
| Conditional Effect:  High symptoms | 2.10, 9.00, .23, <0.001 | 1.84, 7.93, .23, <0.001 | 1.50, 6.96, .21, <0.001 |
| Intermediate symptoms | 1.59, 8.25, .19, <0.001 | 1.45, 7.14, .20, <0.001 | 1.18, 5.70, .21, <0.001 |
| Low symptoms | 1.18, 4.98, .24, <0.001 | 1.07, 3.38, .32, .0007 | .86, 2.73, .31, .006 |
| **Physical bodily threat**  Interaction: | n.s. | n.s. | n.s. |
| **Sexual harassment**  Interaction: | .71, 2.54, .28, .01 | .51, 3.07, .17, .002 | n.s. |
| Conditional Effect:  High symptoms | 1.65, 5.68, .29, <0.001 | 1.46, 5.51, .26, <.001 |  |
| Intermediate symptoms | 1.16, 5.27, .21, <0.001 | .81, 3.51, .23, .0005 |  |
| Low symptoms | .74, 2.72, .27, .007 | .16, .45, .36, .65 |  |
| **Sexual abuse**  Interaction: | 1.40, 4.20, .33, <0.001 | .54, 2.66, .20, .008 | n.s. |
| Conditional Effect:  High symptoms | 2.04, 5.84, .35, <0.001 | 1.58, 4.66, .34, <.001 |  |
| Intermediate symptoms | 1.05, 3.80, .27, .0001 | .90, 3.16, .28, .002 |  |
| Low symptoms | .24, .68, .35, .49 | .21, .49, .42, .62 |  |

|  |
| --- |

Process, moderation analysis. n.s. = non-significant

**Table S2.** Psychotic symptoms as a moderator between alcohol use and subtypes of trauma

|  | **Psychotic symptoms** |
| --- | --- |
|  | ß, t, se, p |
| **Emotional neglect**  Interaction: | n.s. |
| **Emotional abuse**  Interaction: | .39, 2.44, .16, .01 |
| Conditional Effect:  High symptoms | .81, 5.96, .14, <.001 |
| Intermediate symptoms | .43, 2.63, .16, .008 |
| Low symptoms | .43, 2.63, .16, .008 |
| **Physical abuse**  Interaction: | .79, 3.23, .24, .001 |
| Conditional Effect:  High symptoms | 2.11, 8.84, .24, <.001 |
| Intermediate symptoms | 1.32, 4.80, .27, <.001 |
| Low symptoms | 1.32, 4.80, .27, <.001 |
| **Physical bodily threat**  Interaction: | .53, 2.55, .21, .01 |
| Conditional Effect:  High symptoms | 1.20, 6.57, .18, <.001 |
| Intermediate symptoms | .66, 3.09, .21, .002 |
| Low symptoms | .66, 3.09, .21, .002 |
| **Sexual abuse**  Interaction: | .78, 2.01, .39, .04 |
| Conditional Effect:  High symptoms | 2.69, 7.73, .35, <.001 |
| Intermediate symptoms | 1.91, 4.73, .41, <.001 |
| Low symptoms | 1.91, 4.73, .41, <.001 |

**Table S3.** Mental health symptoms as a moderator between tobacco use and subtypes of trauma

|  | **Psychotic symptoms** |  | **Dysfunctional personality** |  |
| --- | --- | --- | --- | --- |
|  | ß, t, se, p |  | ß, t, se, p | |
| **Emotional neglect**  Interaction: | n.s. |  | n.s. | |
| **Emotional abuse**  Interaction: | .31, 2.64, .11, .008 |  | .19, 2.43, .08, .02 | |
| Conditional Effect:  High symptoms | .34, 3.34, .10, .0008 |  | .37, 3.12, .12, .002 |  |
| Intermediate symptoms | .03, .23, .12, .81 |  | .15, 1.59, .10, .11 |  |
| Low symptoms | .03, .23, .12, .81 |  | -.07, -.48, .14, .64 |  |
| **Physical abuse**  Interaction: | .69, 3.76, .18, .002 |  | .36, 2.41, .15, .02 |  |
| Conditional Effect:  High symptoms | .86, 4.23, .20, <.001 |  | .66, 3.23, .20, .001 |  |
| Intermediate symptoms | .38, 2.21, .17, .03 |  | .24, 1.33, .18, .18 |  |
| Low symptoms | -.02, -.13, .21, .90 |  | -.17, -.60, .29, .55 |  |
| **Sexual abuse**  Interaction: | .83, 2.84, .29, .005 |  |  |  |
| Conditional Effect:  High symptoms | -.63, -2.07, .30, .04 |  |  |  |
| Intermediate symptoms | -.15, -.63, .24, .53 |  |  |  |
| Low symptoms | 1.91, 4.73, .41, <.001 |  |  |  |
|  |  |  |  |  |

**Table S4.** Cannabis use as a moderator between mental health symptoms and subtypes of trauma

|  | **Depressive symptoms** | **Anxiety symptoms** | **Suicide symptoms** |
| --- | --- | --- | --- |
|  | ß, t, se, p | ß, t, se, p | ß, t, se, p |
| **Emotional neglect** Interaction: | -.01, -1.74, .003, .08 | -.005, -1.67, .002, .09 | .01, 2.59, .002, .010 |
| Conditional Effect:  High symptoms | .23, 10.39, .02, <0.001 | .20, 9.61, .02, <0.001 | .015, 8.12, .02, <0.001 |
| Intermediate symptoms | .26, 15.07, .02, <0.001 | .22, 14.01, .02, <0.001 | .12, 8.20, 01. <0.001 |
| Low symptoms | .27, 14.21, .02, <0.001 | .23, 13.23, .02, <0.001 | .10, 6.41, .02, <0.001 |
| **Emotional abuse**  Interaction: | n.s. | n.s. | .01, 2.91, .003, .004 |
| Conditional Effect:  High symptoms |  |  | .15, 7.20, .02, <0.001 |
| Intermediate symptoms |  |  | .11, 6.59, .02, <0.001 |
| Low symptoms |  |  | .09, 4.92, .02, <0.001 |
| **Physical abuse**  Interaction: | -.01, -2.49, .01, .01 | -.01, -1.77, .004, .08 | n.s. |
| Conditional Effect:  High symptoms | .18, 4.21, .04, <0.001 | .17, 4.54, .04, <0.001 |  |
| Intermediate symptoms | .25, 6.25, .04, <0.001 | .22, 6.10, .04, <0.001 |  |
| Low symptoms | .28, 6.27, .04, <0.001 | .24, 5.93, .04, <0.001 |  |
| **Physical bodily threat**  Interaction: | -.01, -2.15, .004, .03 |  | n.s. |
| Conditional Effect:  High symptoms | .06, 1.70, .03, .09 |  |  |
| Intermediate symptoms | .11, 3.79, .03, .0001 |  |  |
| Low symptoms | .13, 4.13, .03, <0.001 |  |  |
| **Sexual harassment**  Interaction: | n.s. | n.s. | n.s. |
| **Sexual abuse**  Interaction: | -.01, -1.79, .01, .07 | n.s. | n.s. |
| Conditional Effect:  High symptoms | .12, 1.95, .06, .05 |  |  |
| Intermediate symptoms | .19, 3.40, .06, .0007 |  |  |
| Low symptoms | .22, 3.56, .06, .0004 |  |  |
|  | **Psychosis symptoms** | **Personality** |  |
|  | ß, t, se, p | ß, t, se, p |  |
| **Emotional neglect**  Interaction: | .004, 1.75, .002, .08, | -.01, -3.11, .003, .002 |  |
| Conditional Effect:  High symptoms | .02, 1.56, .02, .12 | .21, 8.49, .02, <0.001 |  |
| Intermediate symptoms | .01, .44, .01, .44 | .26, 13.84, .02, <0.001 |  |
| Low symptoms | -.004, -.26, .01, .79 | .28, 13.62, .02, <0.001 |  |
| **Emotional abuse**  Interaction: | .01, 3.25, .002, .001 | -.01, -2.01, .004, .045 |  |
| Conditional Effect:  High symptoms | .02, 1.25, .02, .21 | .20, 7.02, .03, <0.001 |  |
| Intermediate symptoms | -.02, -1.21, .01, .23 | .24, 10.59, .02, <0.001 |  |
| Low symptoms | -.03, -2.26, .02, .02 | .26, 10.29, .02, <0.001 |  |
| **Physical abuse**  Interaction: | .01, 2.92, .003, .004 | -.02, -3.48, .006, .0005 |  |
| Conditional Effect:  High symptoms | .06, 1.99, .03, .047 | .22, 4.76, .05, <0.001 |  |
| Intermediate symptoms | .002, .07, .03, .94 | .32, 7.53, .05, <0.001 |  |
| Low symptoms | -.02, -.77, .03, .44 | .37, 7.69, .05, <0.001 |  |
| **Physical bodily threat**  Interaction: | n.s. | n.s. |  |
| Conditional Effect:  High symptoms |  |  |  |
| Intermediate symptoms |  |  |  |
| Low symptoms |  |  |  |
| **Sexual harassment**  Interaction: | .01, 1.95, .004, .05 | -.01, -1.71, .01, .09 |  |
| Conditional Effect:  High symptoms | <0.001, .001, .03, .10 | .22, 4.28, .05, <0.001 |  |
| Intermediate symptoms | -.04, -1.40, .03, .16 | .28, 5.92, .05, <0.001 |  |
| Low symptoms | -.06, -1.82, .03, .07 | .31, 5.81, .05, <0.001 |  |
| **Sexual abuse**  Interaction: | .02, 3.42, .01, .0006 | -.02, -2.51, .01, .01 |  |
| Conditional Effect:  High symptoms | .08, 2.01, .04, .04 | .17, 2.54, .07, .01 |  |
| Intermediate symptoms | -.01, -.23, .04, .82 | .27, 4.56, .06, <0.001 |  |
| Low symptoms | -.05, -1.20, .04, .23 | .32, 4.81, .07, <0.001 |  |

**Table S5.** Alcohol use as a moderator between mental health symptoms and subtypes of trauma

|  | **Psychosis symptoms** | **Suicide symptoms** | **Somatic symptoms** |
| --- | --- | --- | --- |
|  | ß, t, se, p | ß, t, se, p | ß, t, se, p |
| **Emotional Neglect**  Interaction: | n.s. | .01, 3.06, .002, .002 | -.01, -2.63, .003, .009 |
| Conditional Effect:  High symptoms |  | .16, 8.52, .02, <.001 | .24, 9.99, .02, <.001 |
| Intermediate symptoms |  | .12, 8.36, .01, <.001 | .28, 15.56, .02, <.001 |
| Low symptoms |  | .08, 3.92, .02, .0001 | .33, 12.76, .03, <.001 |
| **Emotional abuse**  Interaction: | .01, 3.17, .002, .002 | .01, 3.75, .003, .0002 | n.s. |
| Conditional Effect:  High symptoms | .03, 1.46, .02, .14 | .17, 7.83, .02, <.001 |  |
| Intermediate symptoms | -.01, -1.05, .01, .29 | .11, 6.75, .02, <.001 |  |
| Low symptoms | -.05, -2.81, .02, .005 | .05, 2.27, .02, .02 |  |
| **Physical abuse**  Interaction: | .01, 3.55, .003, .0004 | n.s. | n.s. |
| Conditional Effect:  High symptoms | .07, 2.49, .03, .01 |  |  |
| Intermediate symptoms | -.002, -.08, .03, .94 |  |  |
| Low symptoms | -.07, -1.97, .04, .05 |  |  |
| **Physical bodily threat**  Interaction: | .07, 2.61, .003, .009 | n.s. | n.s. |
| Conditional Effect:  High symptoms | .06, 2.47, .02, .01 |  |  |
| Intermediate symptoms | .01, .73, .02, .46 |  |  |
| Low symptoms | -.03, -1.03, .03, .30 |  |  |
|  | **Depressive symptoms** | **Anxiety symptoms** | **Memory symptoms** |
|  | ß, t, se, p | ß, t, se, p | ß, t, se, p |
| **Emotional Neglect**  Interaction: | n.s. | n.s. | .01, 2.06, .003, 04 |
| Conditional Effect:  High symptoms |  |  | .21, 9.14, .02, <.001 |
| Intermediate symptoms |  |  | .18, 10.09, .02, <.001 |
| Low symptoms |  |  | .15, 5.80, .03, <.001 |
| **Physical abuse**  Interaction: | -.01, -2.07, .05, .04 | -.01, -2,29, .004, .02 | n.s. |
| Conditional Effect:  High symptoms | .17, 4.08, .04, <.001 | .17, 4.26, .04, <.001 |  |
| Intermediate symptoms | .24, 5.97, .04, <.001 | .23, 6.33, .04, <.001 |  |
| Low symptoms | .30, 5.29, .06, <0.001 | .29, 5.66, .05, <0.001 |  |

|  | **Dysfunctional Personality** | |
| --- | --- | --- |
|  | ß, t, se, p | |
| **Emotional Neglect**  Interaction: | n.s. | |
| **Emotional abuse**  Interaction: | n.s. | |
| **Physical abuse**  Interaction: | -.01, -2.55, .01, .01 | |
| Conditional Effect:  High symptoms | .24, 5.16, .05, <.001 | |
| Intermediate symptoms | .32, 7.49, .04, <.001 | |
| Low symptoms | | .41, 6.61, .06, <.001 |

**Table S6.** Tobacco use as a moderator between mental health symptoms and subtypes of trauma

| **Electric** | **Psychosis symptoms** | **Suicide symptoms** | **Memory** |
| --- | --- | --- | --- |
|  | ß, t, se, p | ß, t, se, p | ß, t, se, p |
| **Emotional Neglect**  Interaction: | n.s. | n.s. | n.s. |
| **Emotional abuse**  Interaction | n.s. | n.s. | .01, 2.30, .004, .02 |
| Conditional Effect:  High symptoms |  |  | .25, 8.63, .03, <.001 |
| Intermediate symptoms |  |  | .20, 9.57, .02, <.001  .18, 8.09, .02, <.001 |
| Low symptoms |  |  |  |
|  |  |  |  |
| **Sexual abuse**  Interaction: | .02, 4.19, .01, <.001 | .04, 3.79, .001, .0002 | .03, 2.38, .01, .02 |
| Conditional Effect:  High symptoms | .09, 2.36, .04, .02 | .40, 6.28, .06, <.001 | .45, 5.60, .08, <.001 |
| Intermediate symptoms | -.02, -.71, .03, .48 | .22, 5.15, .04, <.001 | .31, 5.67, .05, <.001 |
| Low symptoms | -.06, -2.06, .04, .04 | .15, 3.31, .05, <.001 | .26, 4.32, .06, <.001 |
| **Sexual harassment**  Interaction: | .02, 4.18, .01, <.001 | n.s. | .02, 2.45, .01, .01 |
| Conditional Effect:  High symptoms | .09, 2.36, .04, .02 |  | .29, 5.03, .06, <.001 |
| Intermediate symptoms | -.02, -.71, 03, .48 |  | .19, 4.46, .04, <.001  .16, 3.33, .05, <.001 |
| Low symptoms | -.06, -2.06, .03, .04 |  |  |
| **Physical abuse**  Interaction: | .02, 4.36, .005, <.001 | n.s. | n.s. |
| Conditional Effect:  High symptoms | .12, 3.76, .03, .002 |  |  |
| Intermediate symptoms | .02, .95, .02, .34 |  |  |
| Low symptoms | -.01, -.44, .03, .66 |  |  |
| **Physical bodily threat**  Interaction: | .01, 3.11, .004, .002 | n.s. | .01, 2.04, .01, .04 |
| Conditional Effect:  High symptoms | .08, 3.27, .03, .001 |  | .22, 5.65, .04, <.001 |
| Intermediate symptoms | .03, 1.46, .02, .14 |  | .16, 5.74, .03, <.001 |
| Low symptoms | .01, .27, .02, .79 |  | .14, 4.60, .03, <.001 |
|  | **Repetition** | **Dysfunctional personality** |  |
|  | ß, t, se, p | ß, t, se, p |  |
| **Emotional Neglect**  Interaction: | n.s. | n.s. |  |
| **Emotional abuse**  Interaction | .01, 2.06, .04, .04 | .01, 2.42, .005, .02 |  |
| Conditional Effect:  High symptoms | .26, 9.91, .03, <.001 | .31, 9.93, .03, <.001 |  |
| Intermediate symptoms | .21, 11.53, .02, <.001 | .25, 11.21, .02, <.001 |  |
| Low symptoms | .21, 10.00, .02, <.001 | .23, 9.57, .02, <.001 |  |
|  |  |  |  |
| **Sexual harassment**  Interaction: | n.s. | .02, 1.98, .01, .05 |  |
| Conditional Effect:  High symptoms |  | .39, 6.18, .06, <.001 |  |
| Intermediate symptoms |  | .30, 6.43, .05, <.001 |  |
| Low symptoms |  | .27, 5.32, .05, <.001 |  |
| **Physical abuse**  Interaction: | .01, 2.08, .006, .04 | n.s. |  |
| Conditional Effect:  High symptoms | .38, 8.83, .04, <.001 |  |  |
| Intermediate symptoms | .32, 9.09, .04, <.001 |  |  |
| Low symptoms | .30, 7.72, .04, <.001 |  |  |
| **Physical bodily threat**  Interaction: | n.s. | .01, 2.01, ,01, .04 |  |
| Conditional Effect:  High symptoms |  | .20, 4.72, .04, <.001 |  |
| Intermediate symptoms |  | .14, 4.49, .03, <.001 |  |
| Low symptoms |  | .12, 3.45, .03, <.001 |  |
| **Cigarettes** | **Psychosis symptoms** | **Anger symptoms** | **Repetition** |
|  | ß, t, se, p | ß, t, se, p | ß, t, se, p |
| **Emotional Neglect**  Interaction: | n.s. | n.s. | .003, 2.25, .002, .02 |
| Conditional Effect:  High symptoms |  |  | .20, 9.17, .02, <.001 |
| Intermediate symptoms |  |  | .16, 9.92, .02, <.001  .13, 6.09, .02, <.001 |
| Low symptoms |  |  |  |
| **Emotional abuse**  Interaction | .004, 3.02, .001, .003 | n.s. | .002, 2.26, .002, .02 |
| Conditional Effect:  High symptoms | .03, 1.39, .02, .16 |  | .26, 9.86, .03, <.001 |
| Intermediate symptoms | -.01, -1.03, .01, .30 |  | .21, 11.05, .02, <.001  .18, 6.91, .03, <.001 |
| Low symptoms | -.05, -2.62, .02, .009 |  |  |
|  |  |  |  |
| **Sexual abuse**  Interaction: | .01, 3.25, .003, .001 | n.s. | n.s. |
| Conditional Effect:  High symptoms | .11, 2.43, .04, .02 |  |  |
| Intermediate symptoms | -.001, -.01, .04, .99 |  |  |
| Low symptoms | -.09, -1.86, .05, .06 |  |  |
| **Sexual harassment**  Interaction: | .09, 3.60, .02, .0003 | n.s. | n.s. |
| Conditional Effect:  High symptoms | .05, 1.37, .04, .17 |  |  |
| Intermediate symptoms | -.04, -1.52, 03, .13 |  |  |
| Low symptoms | -.12, -3.13, .03, .002 |  |  |
| **Physical abuse**  Interaction: | n.s. | n.s. | n.s. |
| Conditional Effect:  High symptoms |  |  |  |
| Intermediate symptoms |  |  |  |
| Low symptoms |  |  |  |
| **Physical bodily threat**  Interaction: | n.s. | n.s. | n.s. |
| Conditional Effect:  High symptoms |  |  |  |
| Intermediate symptoms |  |  |  |
| Low symptoms |  |  |  |
